# The gentamicin-collagen implant and the risk of distant metastases of rectal cancer following short-course radiotherapy and curative resection: the long-term outcomes of a randomized study

**DOI:** 10.1007/s00384-018-3045-3

**Published:** 2018-04-15

**Authors:** Andrzej Rutkowski, Lucyna Pietrzak, Jacek Kryński, Leszek Zając, Mariusz Bednarczyk, Tomasz Olesiński, Marek Szpakowski, Piotr Saramak, Ireneusz Pierzankowski, Piotr Hevelke, Piotr Surowski, Krzysztof Bujko

**Affiliations:** 1 Department of Oncological Gastroenterology, Maria Sklodowska-Curie Oncology Center, W. K. Roentgena 5, 02-781 Warsaw, Poland; 2 Department of Radiotherapy, Maria Sklodowska-Curie Oncology Center, Warsaw, Poland

**Keywords:** Rectal cancer, Preoperative radiotherapy, Metastases, Gentamicin-collagen implant

## Abstract

**Purpose:**

A previous randomized study conducted by our group showed that application of gentamicin-collagen implant (GCI) into the pelvic cavity after total mesorectal excision (TME) reduced the incidence of distant metastases. Therefore, we decided to conduct a confirmatory study.

**Methods:**

Patients with rectal cancer were included in the study if they met the following criteria: adenocarcinoma of the rectum, preoperative short-term radiotherapy (5 × 5 Gy), and WHO performance score 0–1.

**Results:**

One hundred seventy-six patients were randomly assigned either to an experimental group in which GCI was applied (*n* = 81) or to a control group without GCI (*n* = 81). Median follow-up was 80 months. Cumulative incidence of distant metastases at 5 years was higher in the control group compared to the experimental group: 23.5 vs 8.6% (HR 2.4 [95% CI 1.1–5.5], *P =* 0.005). Overall survival (OS), disease-free survival (DFS), and cancer-specific survival (CSS) did not differ between the experimental group and the control group: HR 0.95 [95% CI 0.55–1.70], *P =* 0.864; HR 0.85 [95% CI 0.50–1.45], *P =* 0.548, and HR 0.5 [95%CI 0.22–1.22], *P =* 0.093, respectively. The predefined by the protocol subgroup analysis for yp stage III disease showed better DFS in the experimental group compared to the control group; HR 0.47 [95%CI 0.23–0.97], *P =* 0.042).

**Conclusions:**

The results confirmed our previous finding that GCI applied in the pelvis significantly reduced the rate of distant metastases in patients after radical rectal cancer resection.

**Electronic supplementary material:**

The online version of this article (10.1007/s00384-018-3045-3) contains supplementary material, which is available to authorized users.

## Introduction

The use of neoadjuvant radiotherapy in rectal cancer reduces the rate of local recurrence but does not improve overall survival [1, 2]. The rate of distant metastases is much the same regardless of preoperative radiotherapy use. The meta-analysis showed no benefit of postoperative 5-fluorouracil-based chemotherapy in terms of overall survival and incidence of distant metastases in patients with rectal cancer after preoperative radiotherapy [3].

Our previous study randomly allocated 229 of 245 consecutive patients scheduled for rectal cancer resection either to local application of gentamicin-collagen implant (GCI) into the pelvic cavity after total mesorectal excision (TME) or to the control group without GCI [4]. The primary endpoint of the study was the rate of postoperative complications. Patients’ characteristics were well balanced between the two treatment-assigned groups. Preoperative short-course radiotherapy or chemoradiation was used in 51% of patients. Application of GCI was associated with reduced postoperative morbidity and, unexpectedly, also with the improvement of overall survival (*P* = 0.004) and disease-free survival (*P* = 0.007) in patients after R0 resection, mainly by reducing the incidence of distant metastases; crude rates were 10 vs 28%; *P* = 0.002. This effect was observed mainly in patients with pathological stage III cancer. Although our first trial already gave statistically and clinically significant results, the answer of whether the application of a gentamicin-collagen sponge had influence on the risk of cancer recurrence (primarily the local recurrence) was the secondary aim of this study. Therefore, the long-term results came as a surprise to us. However, there are some limitations of the study that deserve consideration. Firstly, it was an unplanned analysis of distant recurrence. We hypothesized that GCI reduced the risk of postoperative complications and local recurrence but not distant recurrence. Secondly, we offered participation in this study to all patients regardless of the TNM stage (including stage IV). Analysis of pTNM I–III subgroups was unplanned. Moreover, a search of the literature did not reveal any studies examining the correlation between the use of locally active antibiotics and risk of distant metastasis in rectal cancer. Finally, we decided to conduct a confirmatory randomized trial because type I error might occur (false positive results). The results of this trial showing postoperative complications were published elsewhere [5]. The aim of this article is to present long-term results.

## Methods

The main objective of the study was the comparison of the rate of distant recurrence in patients after R0–1 resection in relation to the GCI application; the subgroup analyses with respect to the pathological stage of disease were predefined by the protocol. The secondary endpoint was the rate of postoperative complications.

This single-institution trial was approved by an ethics committee. Before treatment patients underwent colonoscopy or rectoscopy, CT of the abdomen and pelvis, chest X-ray, blood CEA level, and blood count. Pelvic MRI was not routinely performed because of long waiting list. Decisions on treatment were made at multidisciplinary meetings. Inclusion criteria were as follows: biopsy-proven adenocarcinoma of the rectum located up to 12 cm from the anal verge, cT3–4 or N-positive category, M0, preoperative short-course radiotherapy with 5 × 5 Gy, age ≥ 18 years, WHO performance score 0–1, leukocytes ≥3.5 × 10^9^ /L, neutrophils/granulocytes ≥ 1.5 × 10^9^/L, and hemoglobin ≥ 9.0 g/dL and signed informed consent. A short-course radiotherapy was used as the entry criterion, because at the time of recruitment to the study, such treatment for the patients with resectable rectal cancer with clinical T3–4 or N-positive category was the standard in our institution. The exclusion criteria were as follows: other synchronous primary cancer, allergy to gentamicin or collagen, pregnancy, or concomitant disorders such as ulcerative colitis or Crohn’s disease.

### Preoperative radiotherapy

All patients were treated with 25 Gy in five fractions delivered in 5 days. The technique of preoperative irradiation was described previously [5]. Surgery was performed within 6 days after the completion of radiotherapy. Intraoperative radiotherapy was not used.

### Randomization

Randomization was carried out after radiotherapy and before surgery by telephone to the independent trial office. The eligible patients were randomly assigned 1:1 either to the experimental group in which GCI was applied or to the control group without GCI. Balanced randomization lists were used. No stratification was made.

### Operative details

Patients were operated on after mechanical bowel preparation and systemic antibiotic prophylaxis (intravenous injections of metronidazole 500 mg and cefuroxime 1500 mg three times a day). The surgical procedure was performed through a midline laparotomy incision. Tumors were resected by sharp dissection under direct vision, keeping the fascia propria of the mesorectum intact in accordance with the principles of TME technique described by Heald et al. [6]. Subtotal mesorectal excision with a wide lateral excision including the mesorectal fascia down to minimum 5 cm below the tumor was performed in cancers above 10 cm from the anal verge. A lateral pelvic lymphadenectomy was not performed. Colonic pouches or protective diverting stoma were constructed according to the discretion of the surgeon. Low anterior resection was defined as a resection with anastomosis within 6 cm from the anal verge. In patients requiring an abdominoperineal excision (APE), the prone position and the extralevator type of resection were carried out. In patients randomly assigned to the experimental group, two GCI (Garamycin®; sponge 10 × 10 × 0.5 cm containing 130 mg of gentamicin) were applied in the space created after mesorectal resection. The implants were not wetted before implantation, and the abdominal cavity was washed before GCI application. In the case of APE, the implants were inserted via the perineal wound. Postoperative complications were categorized according to the Clavien-Dindo classification [7].

### Pathological assessment

The standard pathological technique was used. The quality of TME was assessed in the three-stage grading system based on macroscopic examination of resection specimen according to the definitions described previously by Nagtegaal and Quirke [8, 9]. The circumferential resection margin was measured microscopically. The pathological stage was assessed according to the UICC TNM classification (7th edition).

### Adjuvant therapy

Adjuvant chemotherapy was typically given to patients with the ypN1–2 stage, and patients with other poor prognostic factors. Delivering of postoperative chemotherapy and its schedule was left to the discretion of the clinical oncologist.

### Follow-up

Patients were followed at 3-month intervals for 2 years and then 6-month intervals to complete 5 years of observation. Evaluations consisted of physical examination and measuring of blood CEA levels at each visit. The CT of the pelvis and abdomen and the chest X-ray were performed annually and additionally if the CEA level was abnormal. Colonoscopy was recommended 3 and 5 years after treatment. Local recurrence (LR) was defined as evidence of a tumor within the pelvis, anastomosis or in the perineum. Distant recurrence (DR) was defined as evidence of a tumor in any other area. In any case, where the nature of local or distant tumor was uncertain, a biopsy was recommended.

### Statistical analysis

The calculation of the sample size was based on the data from our previous study [4]. It was assumed that the crude rate of distant metastases after curative resection of rectal cancer in stage II–III after a median follow-up of 3 years is 34%. One hundred seventy-six patients with the minimal observation of 3 years were needed to detect a 20% of absolute reduction with GCI, using a two-sided test with a significance level of 0.05 and 80% power.

All analyses were carried out according to the intention-to-treat principle. Differences in categorical data were assessed using χ^2^ test or Fisher’s exact test. Time intervals were calculated from the date of surgery. Distant and local failures were analyzed in the framework of competing risks methods. For distant metastases, local failure or death was taken as a competing risk, while for local failure, distant metastases or death were treated as competing for risk. For comparison of cumulative incidence function (CIF) curves, Grey test was used. To analyze the impact of the cancer stage on the distant metastasis risk, competing risk regression model was applied. The “cmprsk” package for R statistical program was used for competing for risk analysis [10, 11]. Overall survival (OS) and disease-free survival (DFS) were estimated using the Kaplan-Meier method and compared with log-rank test. For calculations of CSS, the only event was death in patients with recurrence; for DFS, the event was death or recurrence.

## Results

**Fig. 1:**
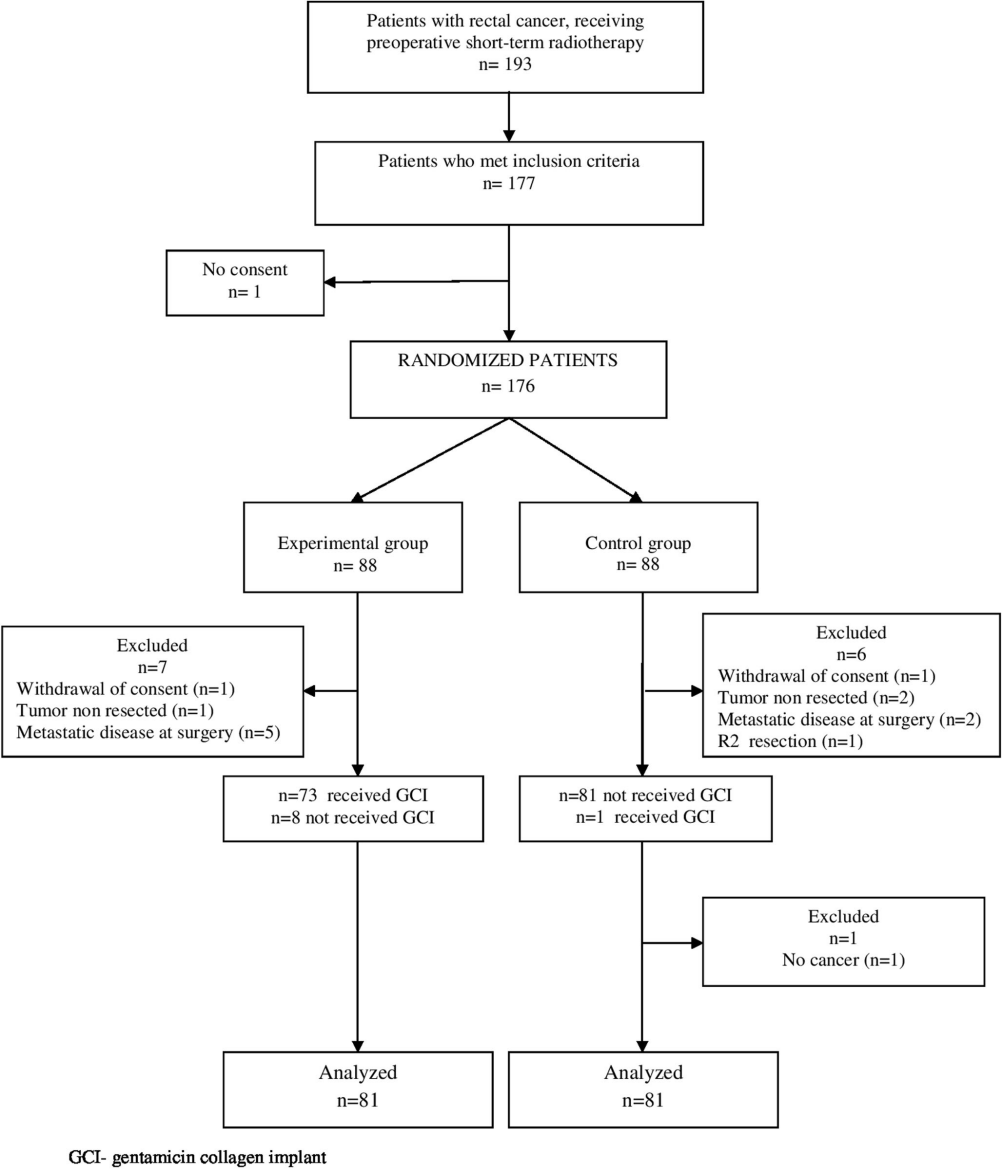
Flow diagram of the study

From January 2008 till September 2011, 176 patients were randomly assigned to the treatment or experimental group. Fourteen patients were excluded (Fig. 1), leaving 162 patients for analysis: 81 in the experimental group and 81 in the control group. Fourteen patients from the experimental group and 12 from the control group were operated within 6–8 weeks after the end of radiotherapy due to respiratory tract infections or for logistic reasons. The remaining patients were operated within a week after radiotherapy. Patients in both groups were well balanced with respect to the pre-treatment characteristics (Table 1). Median follow-up for living patients was 80 months (IQR 68–94) and it did not differ between the groups (*P =* 0.97). None of the patients was lost to a 5-year follow-up. Pathological stage of disease, R1 resection rate, and quality of mesorectal resection did not differ between the groups. About one third of patients from each group received postoperative chemotherapy (35.8% in the experimental group vs 34.6% in the control group; *P* = 0.862). The adjuvant chemotherapy with oxaliplatin was used in 27 patients (33.3%) from the experimental group and in 28 (34.6%) from the control group.

**Table 1:** Characteristics of the patients

	Experimental group *n* = 81 (%)	Control group *n* = 81 (%)	*P* value
Sex			0.402
Male	52 (64.2)	57 (70.4)	
Female	29 (35.8)	24 (29.6)	
Age			0.809
Median (years) (IQR)	63 (57–71)	64 (56–73)	
WHO performance status:			0.622
0	30 (37.0)	27 (33.3)	
1	51 (63.0)	54 (66.7)	
Median distance between anal verge and distal tumor border in cm (range)	5 (1–12)	5 (0–12)	0.992
cT			0.310
1	0 (0)	0 (0)	
2	0 (0)	2 (2.5)	
3	79 (97.5)	78 (96.3)	
4	2 (2.5)	1 (1.2)	
cN			0.550
Negative	21 (25.9)	22 (27.2)	
Positive	53 (65.4)	56 (69.1)	
Unknown	7 (8.6)	3 (3.7)	
CEA level:			0.606
Median (ng/ml)	2.8	2.6	
(IQR)	(1.4–6.2)	(1.45–6.05)	
Type of surgery:			0.526
Abdominoperineal resection	26 (32.1)	23 (28.4)	
Low anterior resection	31 (38.3)	35 (43.2)	
Anterior resection	18 (22.2)	13 (16.0)	
Hartmann’s procedure	6 (7.4)	10 (12.3)	
Intraoperative complications			0.430
Yes+	6 (7.4)	10 (12.3)	
No	75 (92.6)	71 (87.7)	
Postoperative complications#			0.122
Grade I—II	10 (12.3)	20 (24.7)	
Grade III—IV	11 (13.6)	8 (9.9)	
Grade V	0 (0)	0 (0)	
No complications	60 (74.1)	53 (65.4)	
ypT			0.566
0	3 (3.7)	1 (1.2)	
1	1 (1.2)	1 (1.2)	
2	28 (34.6)	21 (25.9)	
3	48 (59.3)	56 (69.1)	
4	1 (1.2)	2 (2.5)	
ypN			0.076
0	49 (60.5)	45 (55.6)	
1	25 (30.9)	19 (23.5)	
2	7 (8.6)	17 (21.0)	
Stage			0.075
0	3 (3.7)	1 (1.2)	
I	22 (27.2)	19 (23.5)	
II	24 (29.6)	25 (30.9)	
III	32 (39.5)	36 (44.4)	
Histological grade			0.277
G1	3 (3.7)	1 (1.2)	
G2	14 (17.3)	22 (27.2)	
G3	6 (7.4)	3 (3.7)	
GX*	58 (71.6)	55 (67.9)	
Quality of mesorectal resection^			0.519
1 (poor)	12 (18.8)	7 (10.9)	
2 (moderate)	6 (9.4)	6 (9.4)	
3 (good)	46 (71.9)	51 (79.7)	
No data	17	17	
Circumferential resection margin			0.719
≤ 1 mm	5 (6.3)	3 (3.8)	
> 1 mm	74 (93.7)	75 (96.1)	
No data	2	3	

*IQR* interquartile range

^According to the three-stage grading system described by Nagtegaal and Quirke [8, 9]

#According to the Clavien-Dindo classification [7]

*Unknown or assessment impossible owing to the postradiation alterations

+Including tumor perforation

### Recurrences

**Fig. 2:**
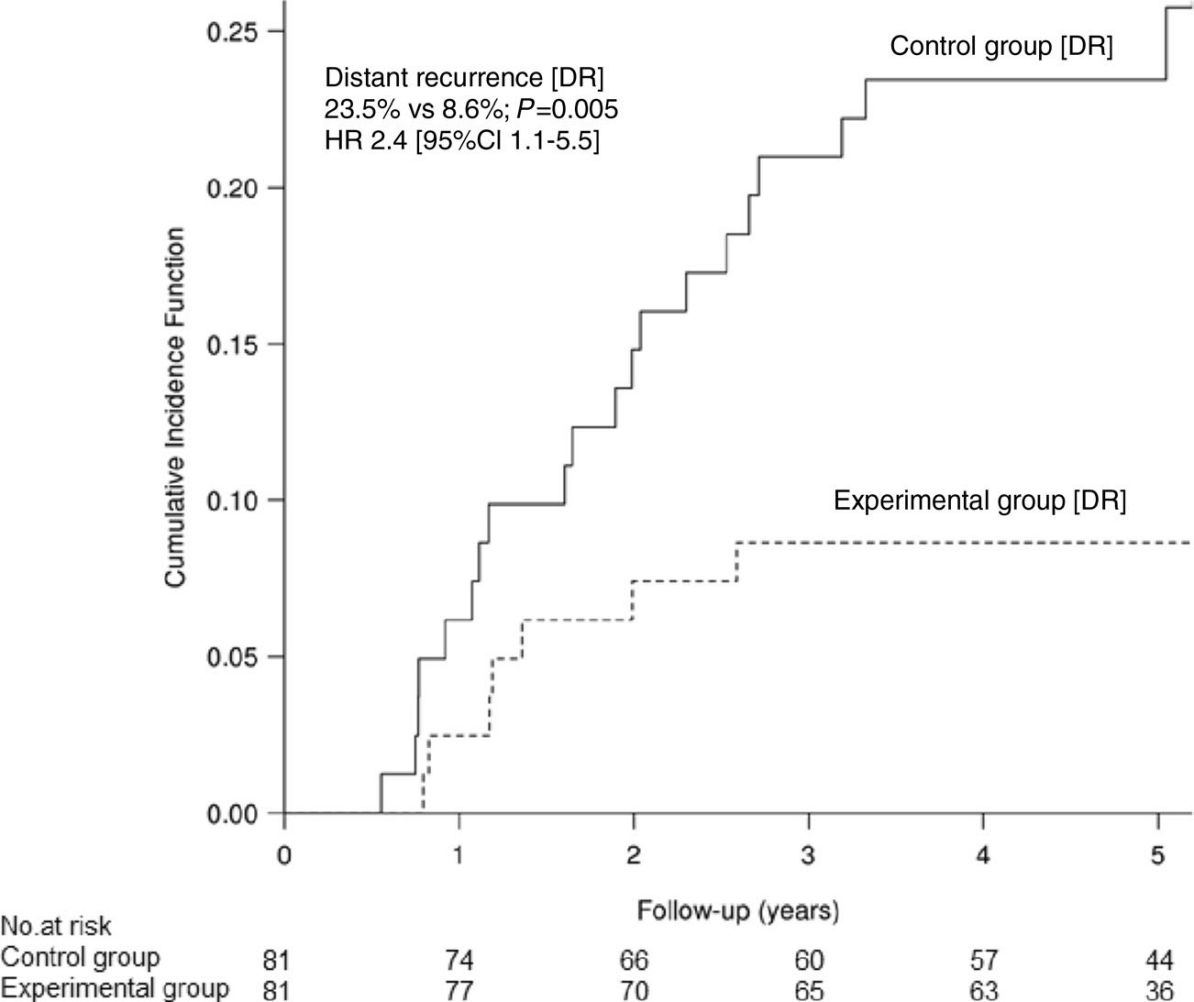
Cumulative incidence of distant recurrence (DR)

The crude rate of distance metastases was 11.1% (*n* = 9) in the experimental group and 24.7% (*n* = 20) in the control group. The five-year cumulative incidence of distant metastases in the control and the experimental group was 23.5% [95% CI 14.2–32.8] vs 8.6% [95% CI 2.5–14.8], respectively (HR 2.4 [95%CI 1.1–5.5], *P =* 0.005)—Fig. 2. The risk of distant metastases in stage III at 5 years was 42.5% [95% CI 25.9–58.8] in the control group vs 16.0% [95%CI 4.3–27.7] in the experimental group. Corresponding rates in stage 0–II were 11.4% [95%CI 3.0–19.8] vs 3.8% [95%CI 0.0–10.6]. Multivariable analysis showed that application of GCI significantly decreased the risk of metastases independently from the disease stage (III vs 0–II): RR 0.43 [95%CI 0.13–0.74], *P =* 0.008—Supplementary material S1. The crude rate of all local recurrence was 9.9% (*n* = 8) in the experimental group vs. 4.9% (*n* = 4) in the control group. Four patients (4.9%) in the control group and three (3.7%) in the experimental group had a local and distant recurrence. The 3-year cumulative incidence of LR was 4.9% [95%CI 0.2–9.7] in the experimental group and 3.7% [95%CI 0.0–7.9] in the control group (*P =* 0.686).

### Survival

**Fig. 3:**
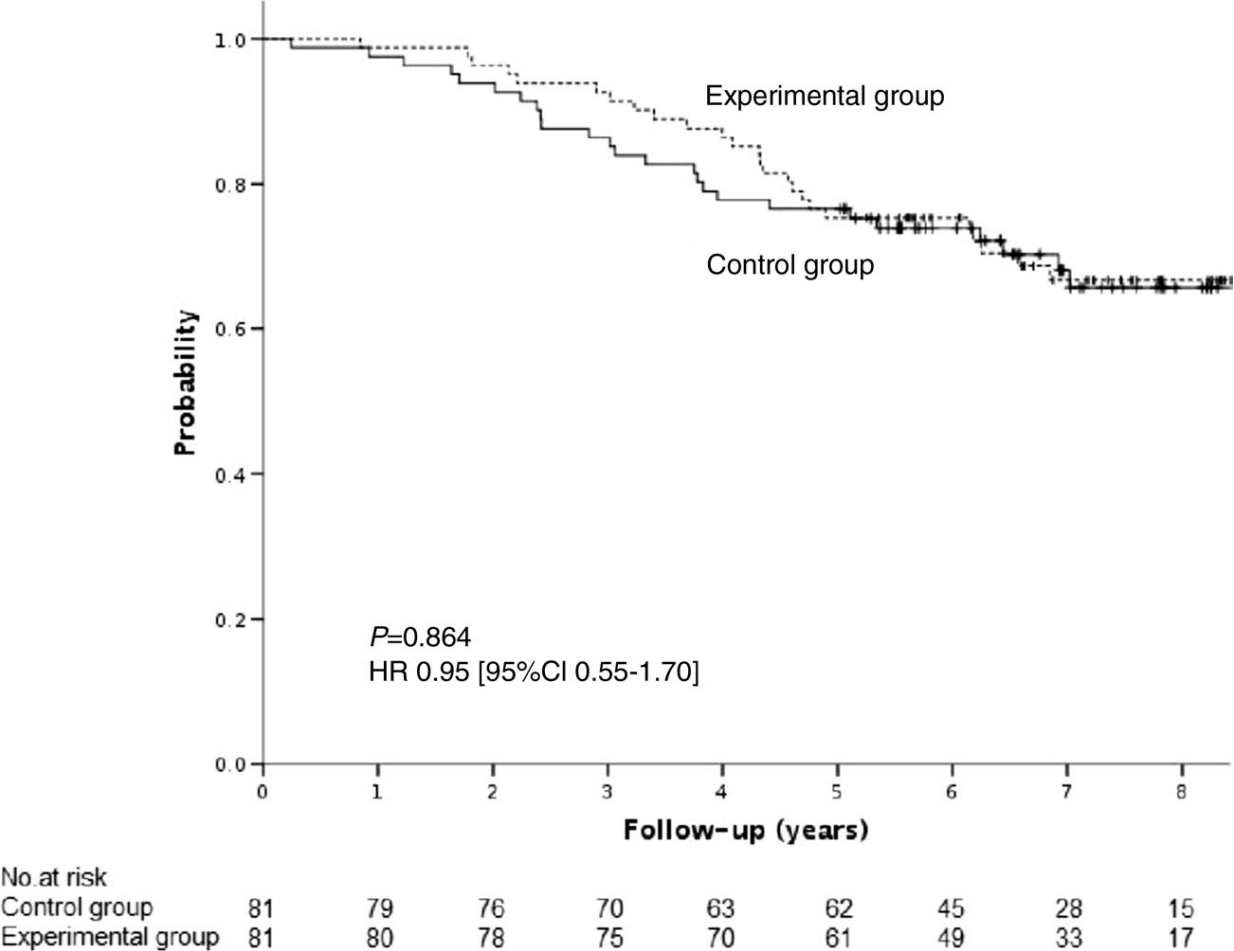
Overall survival (OS)

**Fig. 4:**
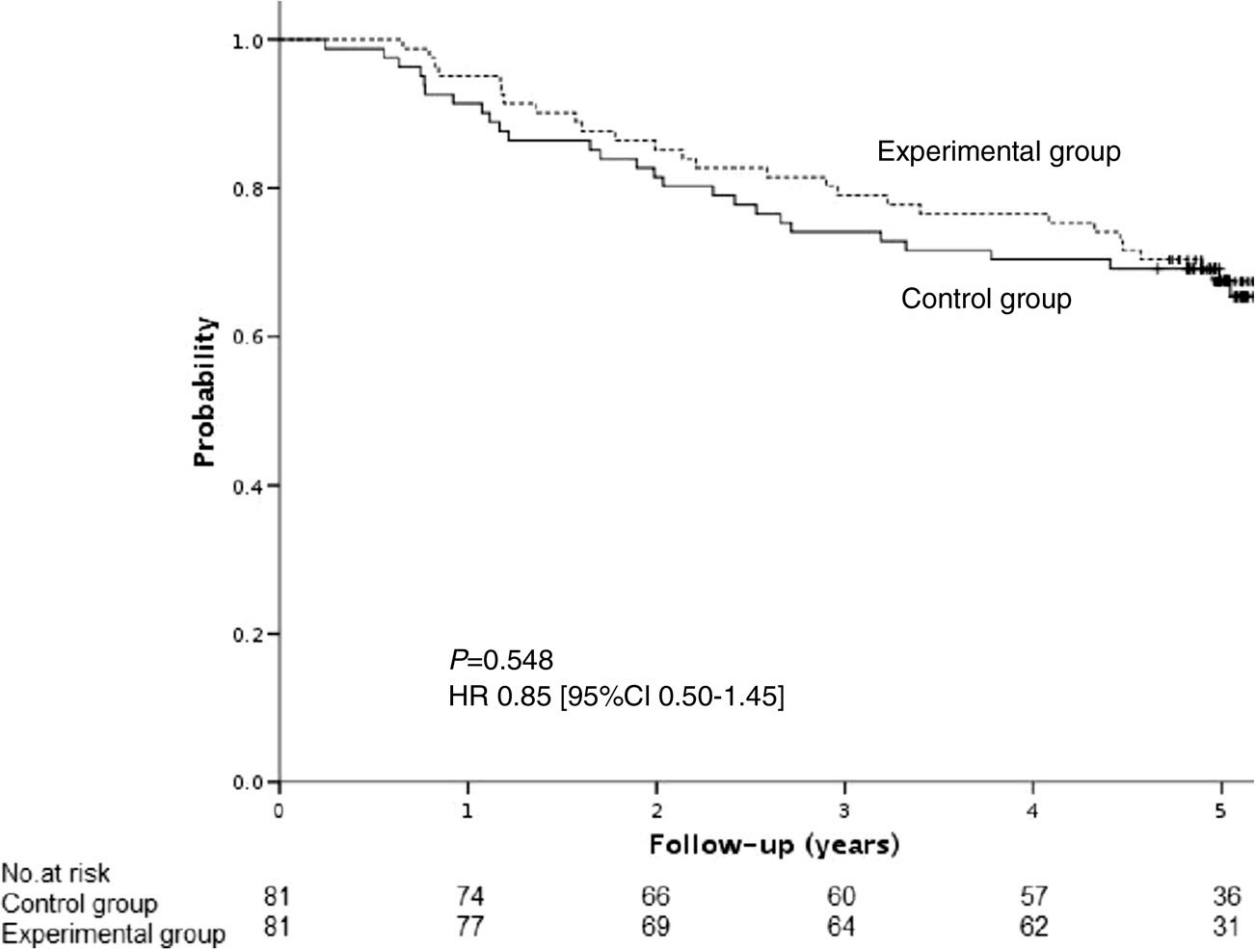
Five-year disease-free survival (DFS)

In the experimental group, 30.9% of patients (*n* = 25) died, including 19.8% (*n* = 16) of patients with intercurrence disease and 11.1% (*n* = 9) of patients with recurrence. In the control group, 30.9% of patients (n = 25) died, including 9.9% (*n* = 8) of patients with intercurrence disease and 21% (*n* = 17) of patients with recurrence. Overall survival (OS) did not differ between the experimental and the control group: HR 0.95 [95%CI 0.55–1.70], *P =* 0.864—Fig. 3. Similarly, cancer-specific survival (CSS) between the two treatment-assigned groups was statistically insignificant: HR 0.5 [95%CI 0.22–1.22], *P =* 0.093. Five-year disease-free survival (DFS) did not differ between the groups: HR 0.85 [95%CI 0.50–1.45], *P =* 0.548—Fig. 4. The difference in DFS was detected between experimental and control group only in patients with yp stage III disease: HR 0.47 [95%CI 0.23–0.97], *P =* 0.042—Supplementary material S2.

## Discussion

The study was designed to detect clinically relevant benefit associated with intraoperative implantation of GCI in order to reduce distant recurrence in a patient after short-term radiotherapy and R0 resection. The results showed that GCI applied in the pelvis significantly reduced the rate of distant metastases (*P =* 0.005).

To date, there have been several clinical studies which have focused on the effects of local application of GCI following rectal cancer surgery. Grüssner and colleagues [12] study focused specifically on 97 patients undergoing abdominoperineal resection. In 49 patients, three GCI (Septocoll®) were inserted into the sacral cavity at one level with the remnants of the levator ani muscle. The results of the study showed that GCI reduced the incidence of postoperative perineal and sacral infections. In a retrospective study conducted by de Bruin et al. [13], three GCI have been inserted in the same place, but another medical product was used (Collatamp®). All patients underwent short-term radiotherapy followed by abdominoperineal resection. The rate of incidence of perineal wound infections and mean hospital stay were significantly reduced in the experimental group. Szynglarewicz and colleagues [14] enrolled 158 consecutive patients, who underwent anterior resection with TME and straight end-to-end anastomosis without a protective stoma. Sixty-five patients received preoperative short-term radiotherapy. All anastomoses were wrapped with GCI (Collatamp®). The rate of anastomotic leakage was 3.2%. In our first randomized study, one GCI (Collatamp®) was placed in the presacral area below the perineal reflection, and when the anterior resection was performed, GCI was wrapped around the anastomosis [4]. In other studies focusing on rectal surgery, the patients with rectal cancer and benign disease were enrolled and the places of implantation of GCI were different [15–18]. Unfortunately, among many trials that investigated GCI in rectal surgery we have found only two, in which oncological outcomes have been recorded (Table 2).

**Table 2:** Literature review

Study	Type of study	No. of patients receiving short-term radiotherapy (%)	Experimental group^ No of DR/No of patients(%)	Control group No of DR/No of patients (%)	*P* value
Nowacki et al. (2004) [4]	Randomized	78 (40.4)	10/97 (10.3)	27/96 (28.1)	0.002
Collin et al. (2012) [19]	Randomized	54 (100.0)	9/26 (34.6)	14/28 (50.0)	0.467
Present study	Randomized	162 (100.0)	9/81 (11.1)	21/81 (25.9)	0.026
Total	–	294 (72.9)	28/204 (13.7)	60/205 (29.3)	< 0.001

*DR* distant recurrence

^Experimental group—gentamicin-collagen implant applied in the pelvic cavity

The first randomized study included 102 patients after abdominoperineal resection [19]. One GCI sponge was placed distal to the levator ani muscle. Only 68 patients underwent surgery for rectal cancer, of which 4 patients had metastatic disease at the time of surgery. One patient was operated for local recurrence, 2 were lost to follow-up, and 7 died due to other causes during a 5-year follow-up. The remaining 54 patients were included in the analysis for cancer recurrence and cancer survival (26 patients in the experimental group and 28 in the control group). There was no difference in the incidence of distant metastases (*P =* 0.467) or local recurrence (*P =* 0.490). However, in the authors’ opinion, the sample size was too small to detect a difference.

The second randomized study conducted in our center enrolled 229 patients (113 in the experimental group and 116 in the control group) [4]. Fourteen patients were excluded. Preoperative radiotherapy was used in 116 (53.2%) patients (81 received short-term radiotherapy, 35—chemoradiotherapy). R0 resection was performed in 193 patients (97 in the experimental group and 96 in the control group). We have noticed the surprisingly low rate of distant recurrence in patients from the experimental group compared to the control group (10.3% vs 28.1%) at 36 months’ follow-up. Moreover, in patients after curative resection (R0; stage I–III), statistically significant difference in overall survival [OS] was recorded *(P =* 0.02). To explain this phenomenon, we had hypothesized that GCI interacted with circulating rectal cancer cells.

Formally, the result of this randomized trial is favorable because the primary endpoint of the study was achieved. We found superiority of GCI use in terms of decreasing the risk of distant recurrence in patients receiving short-term radiotherapy and TME surgery. However, bearing in mind that the rates of distant metastases in the control group were higher, the lack of the difference in OS and DFS between the two treatment-assigned groups is unclear. It could be explained by too small a sample size on the basis of which calculation was prepared to achieve the primary endpoint of the study. Moreover, the mechanism on how GCI might reduce the risk of distant recurrence remains unknown. In the present study, the real-time reverse transcription polymerase chain reaction was used to detect circulating cancer cells in peripheral blood. The samples were taken preoperatively, 24 h, and 7 days after surgery. Unfortunately, we did not find any differences between both randomized groups [20]. Therefore, the hypothesis that GCI interacted with circulating rectal cancer cells was not confirmed. Another option is the correlation between GCI and the radiotherapy in the activation of antitumor immune response. Radiotherapy significantly affect tumor microenvironment with the potential to reverse the immunosuppressive state present in malignancy [21], but the explanations of this effect in the context of the implementation of the GCI are challenging.

Grass and colleagues [22] reported that therapeutic benefits of radiotherapy may not be limited to the tumor volume, but may encompass additional systemic antitumor effects (“abscopal effect”). From the clinical perspective, the term refers to distant tumor regression after localized irradiation. An important role can play daily dose fractionated radiotherapy. Other authors noticed that the most important factor explaining the mechanisms of the abscopal effect is the release of the inflammatory cytokine cascade as a response to ionizing irradiation [23]. Referring to the results of our research, two facts may have a critical role. Firstly, GCI applied into the pelvic cavity after short-term preoperative radiotherapy and TME may reduce the risk of organ-space surgical site infections (SSI) and local inflammatory reactions [5]. Secondly, the positive effect of GCI was observed mainly in a subgroup of patients who underwent surgery immediately after the short-term radiotherapy completion (unplanned analysis—data not shown).

Limitations of the study should be acknowledged. This is a single institutional experience; therefore, obtained results must be treated with caution. Unfortunately, the budget was too small for a multicenter study. Other weaknesses are connected with protocol violations. The quality of TME was not evaluated in 34 (21%) patients. Nine patients were not treated in accordance with the randomization. However, additional (unplanned) per-protocol analysis did not show the difference in relation to the analysis according to the intention-to-treat principle. A further limitation of the study is the imbalance in patients with pathological stage III cancer in the two treatment-assigned groups. This imbalance was not statically significant (*P* = 0.075).

Concluding, our trial suggests a beneficial effect of GCI in terms of decreasing the risk of distant recurrence in patients with rectal cancer receiving short-term radiotherapy and undergoing TME surgery. Further studies are needed to explain the mechanism: how gentamicin-collagen placement might reduce the risk of distant recurrence.

## Electronic supplementary material

ESM 1 (JPEG 250 kb)

ESM 2 (JPEG 171 kb)

ESM 3 (DOC 219 kb)
