# Supplementary figures and images for: The gentamicin-collagen implant and the risk of distant metastases of rectal cancer following short-course radiotherapy and curative resection: the long-term outcomes of a randomized study
Source: Int J Colorectal Dis. 2018 Apr 15;33(8):1087–96. doi: 10.1007/s00384-018-3045-3 (PMC6060799; doi:10.1007/s00384-018-3045-3)

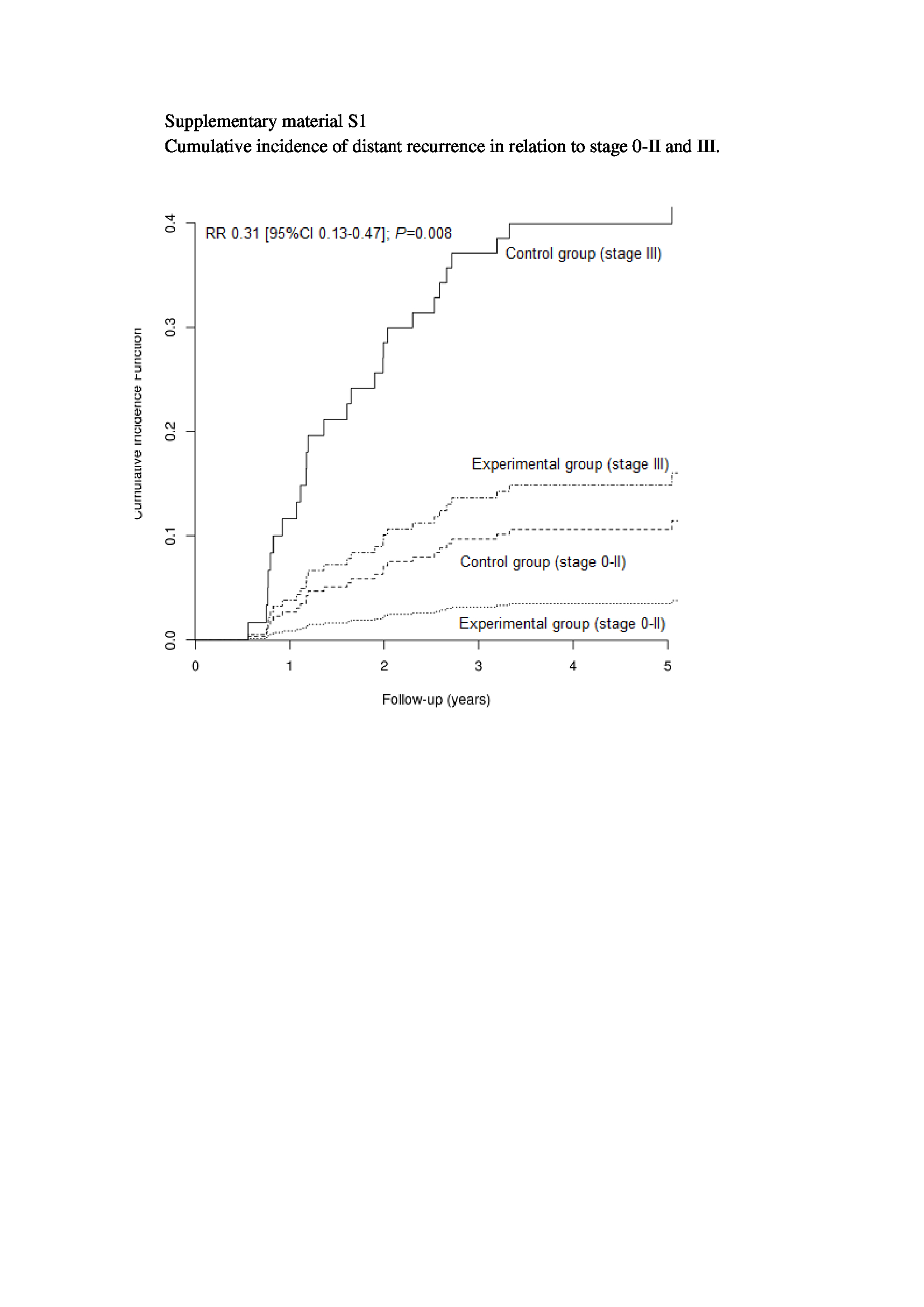

Supplement: Supplementary file 1 — (JPEG 250 kb) [file 384_2018_3045_MOESM1_ESM.jpg]

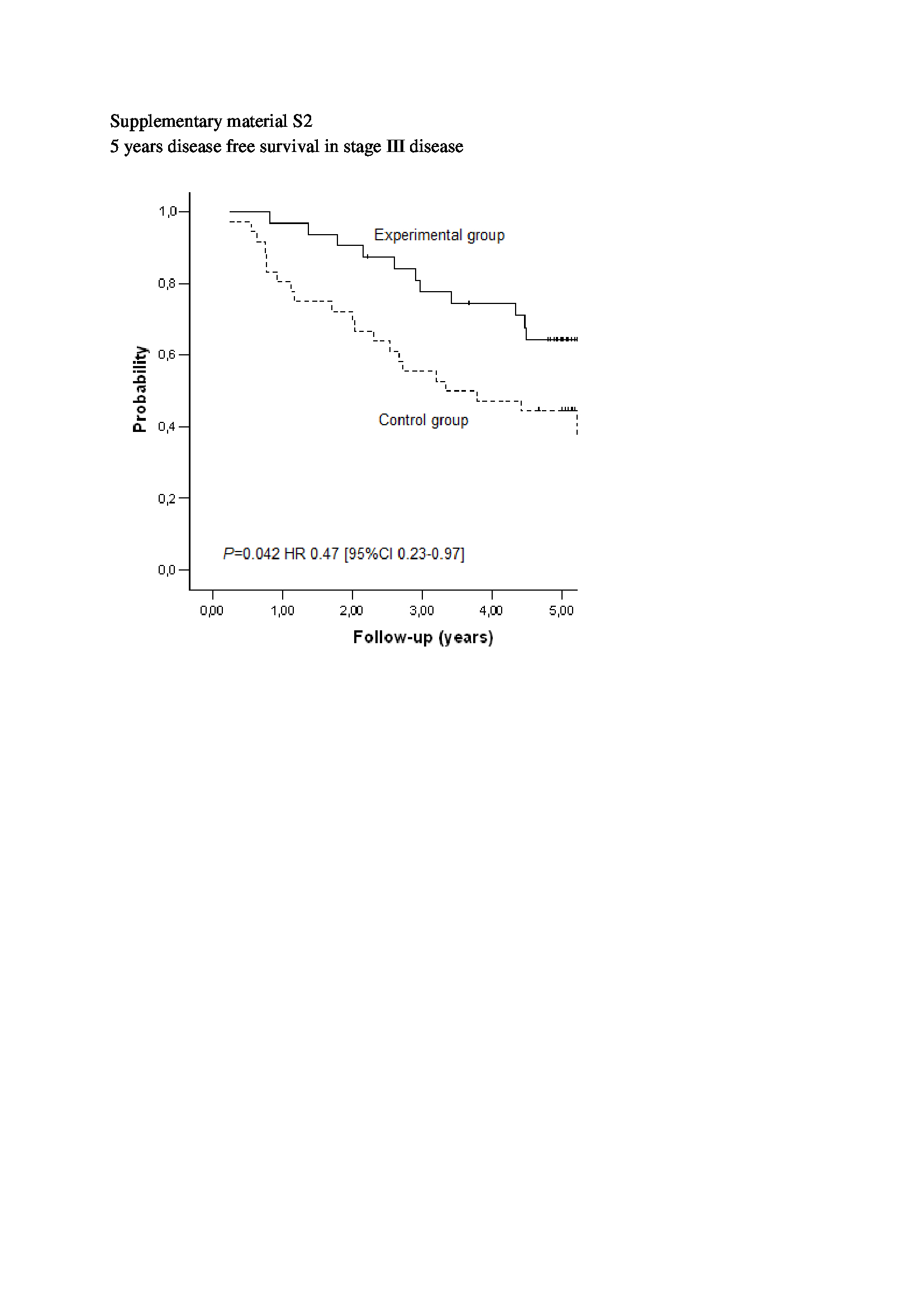

Supplement: Supplementary file 2 — (JPEG 171 kb) [file 384_2018_3045_MOESM2_ESM.jpg]
